# Grenzregion Polen — Deutschland: gestärkt durch die COVID-19-Pandemie

**DOI:** 10.1007/s10273-022-3134-3

**Published:** 2022-03-19

**Authors:** Steffen Fleßa, Julia Kuntosch

**Affiliations:** LS für Allgemeine BWL und Gesundheitsmanagement, Universität Greifswald, Friedrich-Loeffler-Str. 70, 17487 Greifswald, Deutschland

**Keywords:** R10, R11, R12

## Abstract

Die COVID-19-Krise hatte erhebliche Auswirkungen auf die Grenzregion Polen — Deutschland. Der Beitrag untersucht, ob die Pandemie tatsächlich den Abstand zwischen der Grenzregion als strukturschwacher Region und dem Rest von Deutschland erhöhen wird, oder ob die Krise sogar eine Chance bietet. Hierzu wurden neben einer Online-Befragung ebenfalls Experteninterviews durchgeführt und ausgewertet. Die Zielregion ist vergleichsweise gut durch die Krise gekommen, jedoch sollte die Region die Chance nutzen und bereits begonnene Veränderungen wie Digitalisierung und Flexibilisierung weiter vorantreiben.

## Abstract (en)

The COVID-19 pandemic is a humanitarian tragedy with millions of deaths worldwide. However, this virus is also a tremendous economic challenge. There is some evidence that vulnerable groups and regions are in particular at risk of suffering from unemployment, insolvency and other economic hardships during the pandemic. We analyse the economic consequences on one of the poorest regions, the borderland between Germany and Poland. Based on statistical analysis, an online survey of entrepreneurs and key-informant interviews, we can conclude that the consequences of the pandemic are not as disastrous for the region as expected. The instruments of mitigating the economic challenges (including support for enterprises, unemployment insurance and support for temporary under-utilisation of “Kurzarbeitergeld”) were quite successful. The survey partners even see some improvements, in particular in the field of digitalisation. All agree that the flow of information and cooperation between Poland and Germany should have been more intensive to reflect the manifold economic and personal interdependencies between the two countries.

Die menschlichen und medizinischen Folgen der COVID-19-Pandemie sind dramatisch, aber auch die sozioökonomischen Folgen sind gravierend. Die exakte Erfassung ist wahrscheinlich erst mit einem gewissen Abstand möglich, aber erste Schätzungen belaufen sich allein für 2020 auf einen Verlust an weltweiter Wirtschaftskraft von 6,9 Trillionen US-$ (Szmigiera, 2021). Gerade ärmere Länder und Regionen wurden besonders getroffen, was zu einer erheblichen Verschärfung der Armutsquote führen dürfte (Jackson et al., 2021). Auch das Wirtschaftswachstum von 2021 konnte das Defizit von 2020 nicht ausgleichen, und in den folgenden Jahren dürfte die Wirtschaftskraft geringer sein als für eine Entwicklung ohne COVID-19 (Economist, 2021).

Auch in Deutschland sind die Folgen für die Wirtschaft immens. So schätzt das Institut der deutschen Wirtschaft den Wertschöpfungsausfall allein in den Jahren 2020/2021 auf 350 Mrd. Euro (Grömling, 2022). Und auch hier liegt die Vermutung nahe, dass besonders Regionen betroffen sind, die bereits vorher wirtschaftlich schwächer und damit vulnerabler für Krisen waren. So führte der Projektträger Jülich (PTJ) im Auftrag des Bundesministeriums für Bildung und Forschung (BMBF) eine Expertenbefragung zu den „wirtschaftlichen Folgen [der] Corona-Krise in strukturschwachen Regionen“ (April 2020, Oktober 2020, März 2021) durch. Die Fachleute kamen zu dem Schluss, dass „die Gefahr [besteht], dass die strukturschwachen Regionen in der jetzt mit noch mehr Nachdruck verfolgten Transformation der Wirtschaft in Richtung Digitalisierung, Dekarbonisierung sowie in veränderten globalen Wertschöpfungsnetzwerken weiter zurückfallen. Über 80 % der Befragten sehen es als eher oder sehr wahrscheinlich an, dass sowohl die wirtschaftliche Entwicklung, wie auch die Forschungs- und Innovationstätigkeit, in strukturschwachen Regionen mittelfristig stärker als in strukturstarken Regionen in Mitleidenschaft gezogen werden“, sodass „in strukturschwachen Regionen […] die negativen wirtschaftlichen Auswirkungen der Pandemie (Rückgang des BIP, höhere Arbeitslosigkeit, Insolvenzen und anderes) mittelfristig stärker zu spüren sein [werden] als in strukturstarken Regionen Deutschlands“ (König und Kunkel, 2021). Wenn diese Einschätzung zutrifft, dürfte die COVID-19-Krise die Strukturschwäche und wirtschaftliche Schwäche der Grenzregion zwischen Polen und Deutschland in den Bundesländern Brandenburg und Mecklenburg-Vorpommern erheblich verstärken.

## Methodik

Die Studie wurde in gemischter Methodik implementiert. Neben einer unsystematischen Literatur- und Internetrecherche wurden zwei Online-Befragungen und mehrere Experteninterviews durchgeführt. Die Zielregion erstreckt sich über die gesamte deutsche Grenzregion zu Polen und schließt die Kreise der Bundesländer Mecklenburg-

* Die zugrunde liegende Studie wurde als Interreg Kleinstprojekt FMP-0470-21-C gefördert.

**Prof. Dr. Steffen Fleßa** ist Professor für Allgemeine Betriebswirtschaftslehre und Gesundheitsmanagement an der Universität Greifswald.

**Julia Kuntosch** ist dort wissenschaftliche Mitarbeiterin.

Vorpommern (Landkreis Vorpommern-Greifswald) und Brandenburg (Landkreise Uckermark, Barnim, Märkisch-Oderland, Oder-Spree, Spree-Neiße) ein, wobei teilweise auf die vollständigen Bundesländer ausgeweitet wurde. Die Kreise liegen alle unter dem durchschnittlichen BIP pro Einwohner:in, auch die Bundesländer gehören eher zu den ärmeren Regionen (Statistische Ämter der Länder, 2020).

Die Online-Befragung der Unternehmer:innen und Arbeitsagenturen fand im November und Dezember 2021 statt, die Experteninterviews im Januar 2022. Es wurde ein Mixed-Methods-Ansatz gewählt, d. h. die Kombination von quantitativer und qualitativer Forschung mit dem Ziel, neben den Fakten auch die Einschätzungen und Wertungen zu erhalten. Für die Befragung der Unternehmen wurden aus den oben genannten Kreisen alle Unternehmen angeschrieben, die in der Markus-Gewerberegister-Datenbank als Submenge der Orbis-Datenbank vorlagen (6.115). Weiterhin wurden die Geschäftsführungen von 13 Agenturbezirken der Bundesagentur für Arbeit der Bundesländer Mecklenburg-Vorpommern und Brandenburg gebeten, einen entsprechenden Online-Fragebogen auszufüllen. Schließlich wurden fünf Experteninterviews aus Ministerien, Kommunen, Arbeitsagenturen, Unternehmensverbänden und Kammern mit dem Ziel geführt, die quantitativen Ergebnisse der Online-Befragungen in den Gesamtkontext einzuordnen und Lehren zu ziehen.

Es konnten 114 vollständig ausgefüllte Fragebögen zur Auswertung herangezogen werden. Die Teilnehmenden waren bezüglich Branche, Umsatz und Beschäftigtenzahl repräsentativ für die Region. Die Online-Befragung wurde von allen 13 Geschäftsführungen der Arbeitsagenturen in Mecklenburg-Vorpommern und Brandenburg geöffnet, jedoch haben fünf Agenturen sie nicht ausgefüllt. Demnach beziehen sich die weiteren Auswertungen auf die verbleibenden acht Agenturen.

## Auswirkungen der Pandemie

Eine Möglichkeit zur Bewertung der wirtschaftlichen Auswirkungen der COVID-19-Pandemie ist die Zahl der Gewerbean- und -abmeldungen und Insolvenzen. Wie Abbildung 1 zeigt, hat sich die Zahl der An- und Abmeldungen seit Februar 2020 kaum verändert. Auch die Zahl der Unternehmensinsolvenzen ist auf niedrigem Niveau praktisch konstant. Lediglich die Zahl der Privatinsolvenzen (als Differenz der Gesamt- und Unternehmensinsolvenzen) hat während der Pandemie zugenommen, was besonders im ersten Quartal 2021 zu erkennen ist.

Die Pandemie führte in den Unternehmen vor allem zu Kurzarbeit. 95 % der befragten Unternehmen mussten während der Periode 2020/2021 niemanden coronabedingt entlassen,

Abbildung 1
**An- und Abmeldungen sowie Insolvenzen in Mecklenburg-Vorpommern 2020 bis 2021**

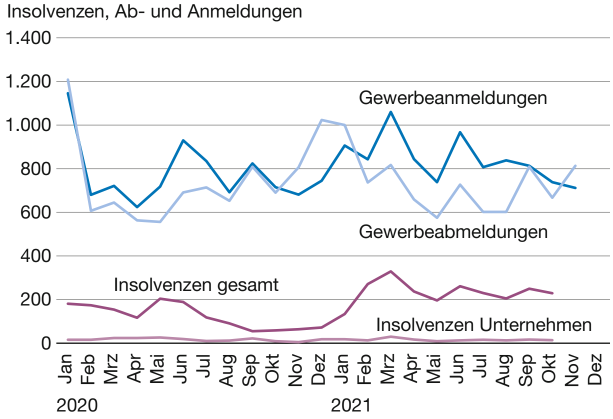

Quelle: Statistische Ämter der Länder (2022).

1 % einen und 4 % zwei Beschäftigte. Hingegen mussten 40 % der Unternehmen Kurzarbeit anmelden, wobei in der intensivsten Periode im Durchschnitt 58 % der Mitarbeitenden von Kurzarbeit betroffen waren.[1] Am häufigsten waren zwischen 40 % und 50 % der Belegschaft in Kurzarbeit.

Dies entspricht den offiziellen Statistiken der Bundesagentur für Arbeit. Sowohl für die Zahl und die Quoten der Arbeitslosen insgesamt, der Arbeitslosen nach SGB II und der Arbeitslosen nach SGB III sowie für die Zahl der gemeldeten Arbeitsstellen führte 2020 tendenziell zu einer Verschlechterung der Situation, die jedoch 2021 wieder ausgeglichen wurde. Ende 2021 ist die Situation vergleichbar oder besser als Ende 2019 (vgl. Abbildung 2 und 3). In einigen Bezirken (z. B. Vorpommern-Greifswald) liegt heute die Arbeitslosigkeit niedriger als vor der Pandemie. Allerdings muss man hier die richtigen Vergleichswerte heranziehen. Schreibt man die positive Tendenz seit 2015 fort, so wäre wohl die Arbeitslosigkeit ohne COVID-19 noch niedriger gewesen. Gleichzeitig ist die Langzeitarbeitslosigkeit in einigen Bereichen tendenziell gestiegen. Die Auswirkungen der Pandemie auf die Wirtschaft in der Grenzregion zwischen Polen und Deutschland sind also nicht so dramatisch gewesen, wie erwartet wurde. Die staatlichen Maßnahmen zur Unterstützung der Wirtschaft und Aufrechterhaltung der Beschäftigung waren anscheinend erfolgreich.

## Maßnahmen

Die Zahl der Maßnahmen zur Unterstützung von Unternehmen während der COVID-19-Pandemie ist hoch. Zum einen sind die Maßnahmen des Bundes (insbesondere

1 In diese Berechnung wurden nur diejenigen Unternehmen einbezogen, die überhaupt Kurzarbeit angemeldet haben.

Abbildung 2
**Arbeitslosenquote jeweils Dezember 2019 bis 2021**

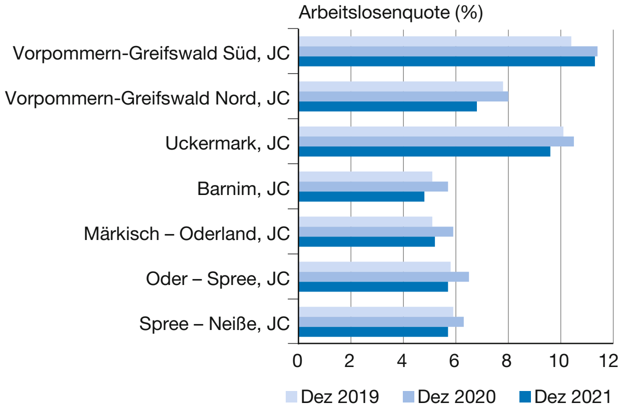

Quelle: Bundesagentur für Arbeit (2022).

Abbildung 3
**Gemeldete Arbeitsstellen Dezember 2019, 2020, 2021**

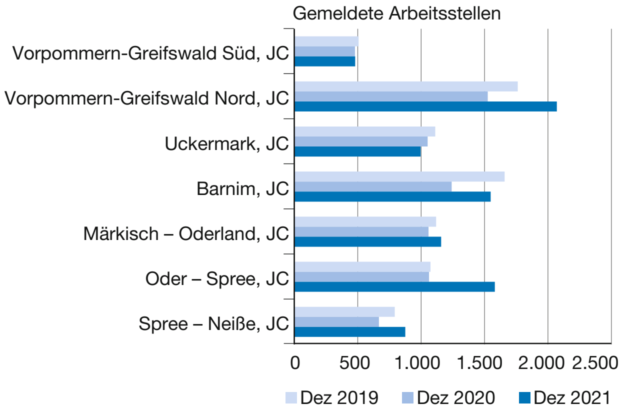

Quelle: Bundesagentur für Arbeit (2022).

Soforthilfe, Überbrückungshilfe I – III Plus, Restart-Prämie, Neustarthilfe), der Bundesländer sowie der Kommunen (Kreise, Städte) zu unterscheiden. Zum anderen kann zwischen Maßnahmen, die bereits vor der Pandemie existierten, und solchen getrennt werden, die speziell hierfür konzipiert und aufgelegt wurden. Ersteres sind vor allem Arbeitslosengeld und Arbeitslosengeld II sowie das Kurzarbeitergeld, wobei die entsprechenden Zugangsregeln zum Teil seit März 2020 vereinfacht wurden.

## Kurzarbeitergeld

Das Instrument, das von allen Interviewpartner:innen, den Agenturen und den Unternehmen als zentral für die Sicherung der Unternehmen eingestuft wird, ist das Kurzarbeitergeld. Vor der Pandemie wurde dieses Instrument vergleichsweise wenig genutzt. So lag die Zahl der Kurzarbeitenden in konjunktureller Kurzarbeit im Agenturbezirk Vorpommern-Greifswald im April 2019 bei 182, im April 2020 jedoch bei 12.573 bzw. bei einer Quote von 15,4 %. Immerhin hatten zu diesem Zeitpunkt 1.989 Betriebe Kurzarbeit angemeldet.

Das Ziel der Kurzarbeit ist es, kurzfristige (konjunkturelle) Beschäftigungstiefs auszugleichen. Der Arbeitgebende zeigt der Agentur für Arbeit die Kurzarbeit an, berechnet und zahlt das Kurzarbeitergeld im Rahmen des Lohnanfalls aus und rechnet dieses anschließend mit der Agentur ab. Die Agentur gewährleistet regelmäßig eine schnelle Bearbeitung, um die Liquidität der Unternehmen nicht unnötig zu belasten. Das Instrument wird von den Verantwortlichen als zentrale Maßnahme der Beschäftigungs- und Unternehmenssicherung bezeichnet. Zur Abmilderung der Pandemie wurden befristet verschiedene Verbesserungen vorgenommen und das Kurzarbeitergeld stufenweise erhöht. Die Kosten beliefen sich bis 31.12.21 auf 42 Mrd. Euro. Der Vorteil für die Arbeitnehmenden liegt auf der Hand, da ihnen die Arbeitslosigkeit erspart bleibt. Aber auch für die Unternehmen und ihre langfristige Arbeitskräftesicherung erweist sich das Instrument als sehr wirkungsvoll. Dass für die Pandemie die Leistungen sogar verbessert wurden, dürfte ebenso ein Anreiz gewesen sein, das Instrument breit zu nutzen.

## Finanzhilfen

Die befragten Unternehmen nutzten die Finanzhilfen nur bedingt und mit zeitlich abnehmender Tendenz. Die Überbrückungshilfe I beantragten gemäß der Befragung der Unternehmen noch 27 %, bei der Überbrückungshilfe II waren es nur noch 10 %, bei der Überbrückungshilfe III 13 %, bei der Überbrückungshilfe III Plus 7 %. Diese Werte entsprechen dem Pandemieverlauf mit einem strikten Lockdown während der Implementierungsperiode von Überbrückungshilfe I, deutlich geringerer Relevanz während der Überbrückungshilfe II, einem erneuten Lockdown während des Zeitraums von Überbrückungshilfe III und geringerer Beschränkung während Überbrückungshilfe III Plus. Die anderen Hilfen wurden nur vereinzelt genannt (z. B. Restart-Prämie zwei Nennungen, Neustarthilfe für Solo-Selbständige eine Nennung, Corona-Schnellkredit KfW zwei Nennungen). Nur eine Person beantragte einen Corona-Unternehmerlohn bei der zuständigen Kommune.

Bei der Befragung der Geschäftsführungen der Agenturen wurden die Instrumente etwas anders bewertet. So schätzen die Befragten die Bedeutung der Instrumente als relativ gleich und konstant ein. Nur die Restart-Prämie und die Neustarthilfe für Solo-Selbständige wurden als weniger bedeutsam deklariert. Als wichtigstes Instrument wurde von den Agenturen das Kurzarbeitergeld eingestuft, was bei der Zielsetzung dieser Institution nicht anders zu erwarten ist.

## Zufriedenheit mit dem Antragsprozess

Die meisten Unternehmen äußerten sich vergleichsweise negativ über den Beantragungsprozess, wobei eine gewisse Tendenz zur steigenden Unzufriedenheit von 2020 zu 2021 erkennbar ist. Besonders bedenklich wurde die administrative Unterstützung durch öffentliche Institutionen eingestuft (vgl. Abbildung 4). Aus Sicht der Interviewten bestand die Schwierigkeit darin, eine Balance zwischen den Zielen „Vermeidung von Missbrauch" und „Praktikabilität für Unternehmen" zu halten. Die Rolle der Steuerberatenden im Antragsprozess wurde ambivalent beurteilt. Einerseits war es gut, dass dadurch ein bundeseinheitliches und digitales Format gewählt wurde, andererseits wurde die Kompetenz der Steuerberatenden zumindest in der Anfangszeit als problematisch eingestuft. Auf jeden Fall eröffnete sich ein neues Geschäftsfeld für Steuerberatende, was von einem Interviewten ebenfalls als Wirtschaftsförderung (für Steuerberatende) deklariert wurde. Zusammenfassend bewerten die Interviewten, dass in den ersten beiden Jahren der Pandemie Geld nicht das primäre Problem gewesen sei, sondern die Komplexität und Schnelligkeit des Antragsverfahrens. Dies ist ein Grund, weshalb die Länder und teilweise die Kommunen eigene, ergänzte Programme aufgelegt haben.

Als Zwischenergebnis hielten die Interviewten übereinstimmend fest, dass die Zielregion erstaunlich gut durch die ersten beiden Jahre der Pandemie gekommen sei. Dieses Ergebnis erstaunt angesichts der peripheren Lage und Wirtschaftskraft der Zielregion. Es stellt sich die Frage, ob die Sondersituation der Grenze zu Polen ein Faktor sein könnte, der in der Analyse der Folgen der Pandemie zu berücksichtigen ist. Deshalb soll im Folgenden die grenzüberschreitende Kooperation diskutiert werden.

## Grenzüberschreitende Kooperation

Seit dem Beitritt Polens zum Schengen-Abkommen am 21. Dezember 2007 haben sich die grenzüberschreitenden Kontakte erheblich intensiviert. Dies betrifft den Handel, die Mobilität von Arbeitnehmenden, die Wahl des Wohnsitzes und Handwerksleistungen. Deutsche Staatsbürger:innen arbeiten zum Teil in Polen, wobei insbesondere die Gravität von Stettin stark zugenommen hat. Aber auch viele polnische Staatsbürger:innen arbeiten in Deutschland und sind hier sozialversichert. Während der ersten drei Wellen der Pandemie wurde die Grenze zwischen Polen und Deutschland für Pendelnde teilweise geschlossen, um eine Ausbreitung von SARS-CoV-2 zu reduzieren. Dies führte speziell für Grenzpendler:innen zu massiven Problemen.

Polnische Staatsbürger:innen, die in Polen wohnen und in Deutschland arbeiten, konnten ihren Arbeitsplatz

Abbildung 4
**Zufriedenheit mit dem Prozess der Maßnahmen**

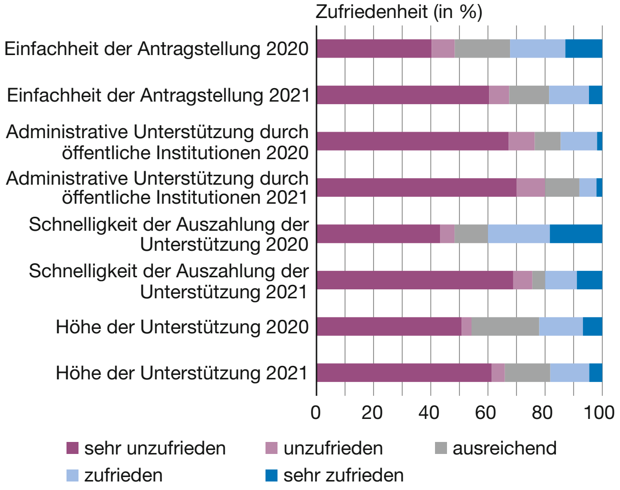

Quelle: eigene Darstellung.

plötzlich nicht mehr erreichen. Insbesondere im Gesundheitswesen führte dies zu Personalengpässen, da viele polnische Ärzt:innen und Pflegekräfte in deutschen Gesundheitseinrichtungen beschäftigt sind und temporär nicht mehr zu Verfügung standen. Während der zweiten und dritten Welle wurde der Nachweis eines aktuellen negativen Schnelltests für den Grenzübertritt obligatorisch, aber der Zeitaufwand war erheblich. In Folge boten manche deutschen Arbeitgebende ihren polnischen Beschäftigten Übernachtungsmöglichkeiten an. Hierfür wurden z. B. von Arbeitgebenden Ferienwohnungen angemietet, sodass die polnischen Beschäftigten nicht mehr täglich pendelten, sondern überwiegend in Deutschland wohnten. Dies verursachte nicht nur Kosten für die entsprechenden Arbeitgeber:innen, sondern war auch für die Beschäftigten ausgesprochen belastend, da sie nicht mehr bei ihren Familien leben konnten.

Grundsätzlich gilt, dass die Unterstützung für Arbeitnehmende und für Unternehmen territorial geregelt ist, d. h., Beschäftigte, die bei Unternehmen in Deutschland sozialversicherungspflichtig angestellt sind, erhalten das volle Portfolio der Unterstützungsmaßnahmen, unabhängig von ihrer Staatsbürgerschaft und dem Ort ihres Arbeitseinsatzes. Polnische Staatsbürger:innen, die an einem deutschen Krankenhaus arbeiten, sind damit genauso in vollem Umfang unterstützungsberechtigt, während deutsche Staatsbürger:innen, die in Polen einer Beschäftigung nachgehen, nur das Arbeitslosengeld II als Grundsicherung aus Deutschland erhalten. Ebenso sind alle Unternehmen förderberechtigt, die in Deutschland angemeldet sind, unabhängig davon, welche Nationalität ihr/e Eigentümer/in hat oder in welchem Land sie überwiegend arbeiten. Gleichzeitig erhalten polnische Unternehmen auch dann keine Unterstützung aus dem deutschen System, wenn sie ausschließlich auf deutscher Seite der Grenze tätig werden.

Betrachtet man zusätzlich den Warenstrom zwischen beiden Ländern, so kann man von einem hochgradig interdependenten gemeinsamen Wirtschaftsraum ausgehen, der durch COVID-19 temporär künstlich getrennt wurde. Das Territorialprinzip der Beschäftigungs- und Wirtschaftsförderung scheint der Realität des Schengenraums zu widersprechen. Umso wichtiger wäre es, die Fördersituation in Polen zu kennen und einschätzen zu können. Die Unternehmen wurden deshalb gefragt, ob ihnen Maßnahmen zur Beschäftigungsförderung während einer Krisensituation bekannt sind, die in Polen eingesetzt wurden. Nur ein Unternehmen bejahte diese Frage, ansonsten herrschte Unkenntnis über die Situation im Nachbarland. Auch die Frage, ob das Unternehmen in einem Austausch mit polnischen Partnern zu den Maßnahmen der Beschäftigungsförderung während der Pandemie stehe, wurde nur von einem Unternehmen bejaht. Es scheint so, dass die deutschen Unternehmen während der Pandemie sich um ihre polnischen Mitarbeitenden gekümmert haben, aber dies führte nicht dazu, dass man sich mit der spezifischen Situation im Nachbarland beschäftigt hat.

Dieselben Fragen wurden den Geschäftsführungen der Arbeitsagenturen gestellt. Eine Geschäftsführung gab an, entsprechende Maßnahmen der Beschäftigungsförderung in Polen zu kennen, aber niemand stand mit polnischen Partnern zu den Maßnahmen der Beschäftigungsförderung während einer Krisensituation in einem direkten Austausch. Die Maßnahmen auf deutscher Seite waren hingegen allen bestens bekannt.

Auf polnischer Seite ist das Maßnahmenportfolio deutlich kleiner als auf deutscher Seite, aber durchaus existent. Bereits im März 2020 wurde der „Anti-Krisenschild“ verabschiedet, um die wirtschaftlichen Folgen der Pandemie abzufangen. Hierzu gehört auch die Möglichkeit, bis zu 40 % des durchschnittlichen Monatsgehalts zuzüglich der Sozialversicherungsbeiträge erstattet zu bekommen. Der „Anti-Krisenschild 2.0“ wurde im Januar 2021 im Umfang von 7,7 Mrd. bis 8,8 Mrd. Euro aktiviert (Deutsch-Polnische Industrie- und Handelskammer, 2020). Dass es in Polen ein Äquivalent zum Kurzarbeitergeld gibt, war keinem Gesprächspartner bekannt.

Die Interviewten wurden gefragt, wie die Zusammenarbeit mit Polen während der Krise eingeschätzt wird. Die überwiegende Antwort ist, dass es zwar einen Informationsaustausch, aber keine Abstimmung untereinander gegeben habe. Es gab auf deutscher Seite Landesprogramme zur Sicherung der Arbeitskräfte im grenznahen Bereich (z. B. Mietzuschuss zugunsten von Grenzpendelnden für Wohnungen auf deutscher Seite, Aufbau von Testzentren). Hierüber wurde die polnische Seite informiert, aber eine gemeinsame Planung wurde nur dort als notwendig erachtet, wo gemeinsame EU-Anträge (REACT bzw. EFRE-Programm) gestellt wurden.

Deutlich stärker war die Zusammenarbeit mit polnischen Partnern der Verbände und Dachorganisationen der Unternehmen. Polen ist für Unternehmen in Mecklenburg-Vorpommern ein sehr wichtiger Handelspartner, wobei sich der Austausch und das Vertrauen über Jahre sehr positiv entwickelt haben. Dieser Wandel beruht auf dauerhaften und respektvollen Beziehungen – eine Vertrauensbasis, die aus Sicht von Interviewten während der COVID-19-Pandemie gelitten habe. *Erstens* wurde Unmut darüber geäußert, dass das Grenzregime am Anfang der Pandemie so strikt war, obwohl die Inzidenz in Polen zu dieser Zeit gering war und keine Gefährdung erkannt werden konnte. Dies habe zu Spannungen mit den polnischen Kollegen und Offiziellen geführt, was die regionale Zusammenarbeit und das Vertrauensverhältnis beschädigt habe.

*Zweitens* wurde berichtet, dass die Kooperations- und Informationsbereitschaft der Landesregierung von Mecklenburg-Vorpommern während der Pandemie nicht so stark gewesen sei, wie sich dies die polnischen Partner gewünscht hätten. Dabei kommt es weniger auf die Frage an, ob dies ein Faktum ist, sondern darauf, ob dies so wahrgenommen wird. Mehrfach wurde in den Interviews betont, dass Wirtschaftsbeziehungen zu den polnischen Partnern darunter leiden könnten, dass sie sich während der Pandemie nicht ausreichend respektiert gefühlt hätten. Diese Wahrnehmung beeinflusst – unabhängig davon, ob dies tatsächlich gegeben ist – die zukünftige Zusammenarbeit.

*Drittens* wurde von mehreren Gesprächspartnern festgestellt, dass die Angemessenheit der digitalen Kommunikation neu zu bewerten sei. Sobald politisch oder kulturell sensible Fragen (z. B. Ausbau des Hafens in Stettin) angesprochen würden, erweise sich die Videokonferenz mit den polnischen Partnern als wenig geeignet, um persönliche Beziehung und Vertrauen aufzubauen. Mehrfach wurde betont, dass das persönliche Gespräch über die Ländergrenze hinweg noch wichtiger sei als zwischen Landsleuten, um Missverständnisse zu vermeiden. Hier hat die Pandemie zu einer Rückkehr der Kulturgrenzen beigetragen.

Zusammenfassend zeigt sich ein ambivalentes Bild. Einerseits waren die Maßnahmen der Coronapolitik primär territorial ausgerichtet und die gegenseitige Information hätte besser sein können. Gleichzeitig fanden Unternehmen, Landesregierungen und Kommunen kreative Wege, um das Beste aus der Situation zu machen. Grenzpendelnde wurden unterstützt, Wirtschaftskontakte wurden weiterentwickelt und die Täler der Coronawellen für den intensiven Austausch genutzt. Man kann aber nicht darauf schließen, dass die Grenznähe die Wirtschaftssituation gestärkt oder geschwächt hätte.

## Bewertung

Abschließend wurden die Geschäftsführungen der Agenturbezirke der Bundesagentur für Arbeit, die Unternehmen sowie die Interviewten gefragt, wie sie die Zukunft „nach Corona" einschätzen und was sie aus der Krise lernen könnten. Abbildung 5 zeigt die Ergebnisse der Befragung der Unternehmen. Nur ein sehr geringer Anteil bezeichnet die eigenen Zukunftsaussichten als gut, über die Hälfte stimmt dieser Bewertung wenig oder gar nicht zu. Dass COVID-19 hierfür mitverantwortlich ist und die bestehenden Probleme des Unternehmens verstärkt hat, glauben mehr als die Hälfte der Befragten. Fast 80 % sind hingegen der Meinung, dass die COVID-19-Finanzhilfen voll und ganz bzw. überwiegend dazu beigetragen haben, dass das eigene Unternehmen die Krise bislang bewältigen konnte. Aber ein weiterer Lockdown könnte nach eigener Einschätzung für viele Unternehmen die Insolvenz bedeuten. Immerhin sehen fast 1/3 der Befragten dies so.

Trotzdem sind sowohl die befragten Unternehmen als auch die Partner:innen der Experteninterviews mit einer großen Mehrheit der Meinung, dass sie während der Coronapandemie sehr viel gelernt hätten, die Krise auch als Chance gesehen werden könne und wir heute besser auf Krisen vorbereitet sein (können) als vor zwei Jahren. Die zentralen Verbesserungen sehen sie in den Bereichen Digitalisierung, Geschäftsmodelle (insbesondere im Einzelhandel), Hygiene, Schnelligkeit, Flexibilität, Resilienz sowie im allgemeinen Umgang mit Krisen.

## Fazit

Wir können schließen, dass die Zielregion bislang relativ gut durch die Krise gekommen ist. Wichtige Wirtschaftsindikatoren sprechen dafür, dass nicht nur die schlimmsten Folgen abgefangen wurden, sondern die wirtschaftliche Situation als stabil bezeichnet werden kann. Zweifelsohne gibt es Branchen und Regionen, die derzeit schwer getroffen sind, aber dies dürfte zu einem gewissen Teil auf strukturellen Ursachen beruhen, die bereits vor Corona existiert haben, jedoch dadurch verstärkt oder beschleunigt wurden. Hier sind besonders periphere Regionen, der Schiffbau und der innerstädtische Einzelhandel zu nennen. Andere Branchen wurden zwar schwer getroffen (z.B. Hotel- und Gaststättengewerbe), können jedoch eine stabile Nachfrage erwarten, sobald die Pandemie zu Ende geht.

Abbildung 5
**Bewertung der Zukunft durch die Unternehmen**

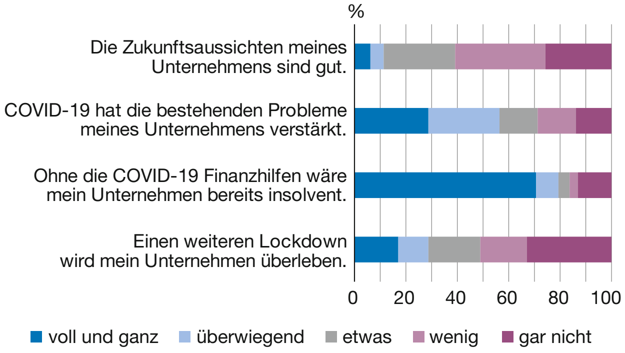

Quelle: eigene Darstellung.

Dennoch sind einige Probleme während der Pandemie offenbar geworden, die zügig gelöst werden sollten. Eines davon ist die Schnelligkeit der öffentlichen Verwaltung. So dauerte nach Aussagen der Befragten die Feinjustierung der Überbrückungshilfen des Bundes zu lange. Hier konnten die Landesverwaltung und die Kommunen zum Teil einspringen, aber die Bürokratie wurde allgemein als große, bereits vor Corona existierende Zukunftsbarriere erkannt. Die Essenz der Aussagen hierzu könnte in folgendem Satz kondensiert werden: „Die Maßnahmen waren gut, aber die Administration könnte schneller sein."

Ein weiteres Problem dürfte die Konzentration auf Territorien sein, die der wirtschaftlichen Realität nicht mehr gerecht wird. Die Verflechtung in den Grenzregionen ist so intensiv, dass unterschiedliche Förderinstrumente und Sozialsysteme der Realität der Unternehmen und Haushalte nicht mehr gerecht werden. Besonders Berufspendelnde in beiden Richtungen hatten erhebliche Nachteile zu erleiden.

Vor allem zeigte sich während der Pandemie erneut die Strukturschwäche der Zielregion. Corona war nicht der Ursprung, wohl aber Beschleuniger und Verstärker von Problemen und Prozessen. Z.B. zeigte die Pandemie, wie dünn die Kapiteldecke der Unternehmen tatsächlich ist. Auch wurde offensichtlich, dass ein auf Effizienz getrimmtes System keine Sicherheitsreserven mehr hat. Die Effizienz verlangt eine möglichst hohe Kapazitätsauslastung, was aber im Krisenfall bedeutet, dass keine Sicherheitsreserven mehr vorhanden sind. Just-in-Time-Lieferketten sind wirtschaftlich, führen aber im Falle einer Krise unmittelbar zu Produktionsausfällen und damit zu gewaltigen Schäden. Auch hier ist COVID-19 nicht der Auslöser, aber die Krise macht den grundlegenden Konflikt zwischen Effizienz und Sicherheit eines Wirtschaftssystems manifest. Es wird die Aufgabe nach der Pandemie sein, diese Erkenntnisse in Wirtschaft und Verwaltung umzusetzen.

Insgesamt überwiegt jedoch der Eindruck einer positiven Entwicklung in der Grenzregion zwischen Polen und Deutschland. Die prognostizierte Schere zwischen Peripherie und Zentrum (König und Kunkel, 2021) hat zumindest in der Grenzregion nicht stattgefunden. Auch der Arbeitsmarkt erwies sich als relativ robust, was zum Teil daran liegt, dass die Wirtschaft überwiegend von KMUs geprägt ist. Mit wenigen Ausnahmen konnten die Betriebe nach den Lockdowns wieder zeitnah durchstarten und zum Teil (z. B. Tourismus) Verlorenes wieder aufholen. Der Anstieg der Arbeitslosigkeit fiel deshalb moderat aus.

Selbstredend ist im dritten Jahr der Pandemie weder ein sanftes Gleichgewicht erreicht, noch sind die Folgen der wirtschaftlichen Erschütterung kurzfristig zu überwinden. Trotzdem zeigt sich, dass die Krise einige Entwicklungen angeschoben hat, die gerade für eine strukturschwache Region zur Initialzündung werden kann. Digitalisierung, Flexibilisierung, Konzentration auf die grenzüberschreitende Verflechtungsregion und enge Kooperation zwischen Unternehmen und mit der Verwaltung sind Innovationen, die in der derzeitigen Erschütterungsphase eine größere Chance haben, sich durchzusetzen als zuvor. Diese Chancen müssen ergriffen werden, denn die Probleme, die schon vor COVID-19 existierten, sind während der Pandemie nicht einfach verschwunden, sondern nur etwas aus dem Fokus geraten. Sie werden wieder sichtbarer werden, wenn die Pandemie weniger aktuell ist.

In der Betriebswirtschaftslehre ist es anerkannt, „dass Unternehmen zum Innovieren Krisen benötigen“ (Perlitz und Löbler, 1985). In der stabilen Phase überwiegt die Vermeidung von Risiken, sodass die bekannten Standardlösungen bevorzugt werden. Notfalls wird mit Subventionen die bestehende Lösung konserviert (Ritter, 2001). In Krisensituationen hingegen ist die Risikobereitschaft höher, die Metastabilisierung des Standards wird unmöglich und der Innovationssprung ist oftmals die einzige Lösung, um aus der Krise zu kommen. Krisen ermöglichen es, verkrustete Strukturen aufzubrechen, Risiken einzugehen, die Zukunft systematisch wertzuschätzen und Freiräume zum Experimentieren zu gewähren. Deshalb stellt die COVID-19-Krise auch für die strukturschwachen Räume entlang der polnisch-deutschen Grenze eine Chance dar, mit Innovation und Zukunftsorientierung neu zu starten. Damit würde die Wirtschaft dieser Region gestärkt aus der Krise hervorgehen. Die Antworten der Studienteilnehmenden lassen keinen Zweifel daran, dass viele bereit sind, diese Chance zu ergreifen und anzugehen.
